# Dental Chemistry—The Dentist's Manual of Special Chemistry

**Published:** 1888-02

**Authors:** 


### Bibliographical.

---

DENTAL CHEMISTRY. THE DENTISTS' MANUAL OF SPECIAL CHEMISTRY.—By Clifford Mitchell, A. B., (Harv.,) M. D. Published by the Author, Chicago, 1887.

This work is intended chiefly for students of dental colleges and attention is paid to detail and careful explanation, which are necessary to those who are commencing the study of dental chemistry. Among the authorities of the author on Physics are Ganot, Deschanelles, Miller, Gage, Avery, Barker, Bartley; on Chemistry, Bloxam, Watts, Wurtz, Gmelin, Roscoe, Allen, Long, Storer, Charles, Ralfe, Simon, Howe and others; to matters chiefly dental his authorities have been Flagg, Essig, Gorgas, Richardson, Black, Wildman, Miller, Allport, Morsman, Harlan, Talbott, A. O. Hunt, Hunter, Gilbert, Abbott, Vergne, Palmer, Brackett and others; for the use of chemical substances in dental medicine, his authority has been the work of Professor Gorgas; and the author states that he is greatly indebted to the latter work for his choice of matter, the chemistry of which he should bring before the dental student.

The first chapter—on Physics and Chemical Physics—is intended chiefly for reference, and to explain many terms used in the body of the work.

The second chapter—on reading and writing formulæ—describes the method used by the author with his own pupils. The remaining chapters comprise compounds of non-metallic elements, of sodium, potassium, calcium, arsenic, &c., metallurgy, alloys, organic chemistry, acids, alkaloids, proteids, ferments, antiseptics, &c., the teeth, calculi and laboratory work, all rendering the volume a very valuable addition to the series of dental text books.

